# Overlapping connectivity patterns during semantic processing of abstract and concrete words revealed with multivariate Granger Causality analysis

**DOI:** 10.1038/s41598-020-59473-7

**Published:** 2020-02-18

**Authors:** Mansoureh Fahimi Hnazaee, Elvira Khachatryan, Sahar Chehrazad, Ana Kotarcic, Miet De Letter, Marc M. Van Hulle

**Affiliations:** 1 Laboratory for Neuro- and Psychophysiology, Department of Neurosciences, KU Leuven, Leuven, Belgium; 2 Numerical Analysis and Applied Mathematics Section, Department of Computer Science, KU Leuven, Leuven, Belgium; 3 Center for the Historiography of Linguistics, Department of Comparative, Historical and Applied Linguistics, KU Leuven, Leuven, Belgium; 4 Medicine and Health Sciences, Department of Rehabilitation Sciences, UGent, Gent, Belgium

**Keywords:** Language, Biophysical models, Network models

## Abstract

Abstract, unlike concrete, nouns refer to notions beyond our perception. Even though there is no consensus among linguists as to what exactly constitutes a concrete or abstract word, neuroscientists found clear evidence of a “concreteness” effect. This can, for instance, be seen in patients with language impairments due to brain injury or developmental disorder who are capable of perceiving one category better than another. Even though the results are inconclusive, neuroimaging studies on healthy subjects also provide a spatial and temporal account of differences in the processing of abstract versus concrete words. A description of the neural pathways during abstract word reading, the manner in which the connectivity patterns develop over the different stages of lexical and semantic processing compared to that of concrete word processing are still debated. We conducted a high-density EEG study on 24 healthy young volunteers using an implicit categorization task. From this, we obtained high spatio-temporal resolution data and, by means of source reconstruction, reduced the effect of signal mixing observed on scalp level. A multivariate, time-varying and directional method of analyzing connectivity based on the concept of Granger Causality (Partial Directed Coherence) revealed a dynamic network that transfers information from the right superior occipital lobe along the ventral and dorsal streams towards the anterior temporal and orbitofrontal lobes of both hemispheres. Some regions along these pathways appear to be primarily involved in either receiving or sending information. A clear difference in information transfer of abstract and concrete words was observed during the time window of semantic processing, specifically for information transferred towards the left anterior temporal lobe. Further exploratory analysis confirmed a generally stronger connectivity pattern for processing concrete words. We believe our study could guide future research towards a more refined theory of abstract word processing in the brain.

## Introduction

Abstract thought and verbal information transfer are two innate cognitive functions of human beings. However, how our brains understand abstract language and how the underlying neural pathways and systems differ from those involved in processing concrete, tangible concepts is not yet clear^1^. Abstract words refer to notions which cannot be touched or sensed, which is why their processing cannot merely rely on the motor and perceptual systems. Experimental data coming from behavioral, neuroimaging (fMRI) and electrophysiological (EEG, MEG) studies of both healthy individuals^2^ and patients suffering from brain disorders^3–5^ show that abstract and concrete words are likely to be processed differently. For example, concrete words have been shown to be learned at an earlier stage of life and understood and retrieved faster^1^. This mechanism is known as the concreteness effect^6,7^. Since it is still unclear how exactly the processes underlying this effect work, various methods and tools have been employed to study them.

Among these methods and tools are neuroimaging techniques such as PET and fMRI which can assess the spatial activation of brain regions during concrete and abstract word processing. These neuroimaging studies have examined a series of hypotheses^8^. For example, one popular hypothesis is that the verbal and nonverbal systems are generally and respectively attributed to the left and right cerebral hemispheres^9^. This idea goes hand in hand with the dual coding theory^10–12^ which states that processing abstract words mainly relies on the verbal while that of concrete words on both verbal and nonverbal systems^9,13^. Accepting these claims, neuroimaging studies suggest that the left hemisphere plays a more prominent role in the processing of abstract and the right hemisphere in that of concrete words. In this respect, some fMRI studies^13–15^ indicate a higher activation in the left temporal and inferior frontal gyri for abstract compared to concrete words and consider these findings to support the dual coding theory. Another well-known theory, the context availability theory, used in similar studies suggests that both abstract and concrete words are processed in one broader contextual system where every word is understood in context^16^. Since the context of abstract words is, unlike that of concrete words, mainly tied to an individual’s experience, their combinations of the underlying neural systems are weighted differently than those of concrete words. Along these lines, several studies have found evidence that seemingly contradicts the pattern predicted by the dual coding theory. Some indicate that greater activation can be observed in the left temporal areas, such as the left basal temporal cortex, for concrete and stronger activations in the right temporal areas for abstract words^17,18^. Others have found greater activity for abstract words in the right hemisphere^7^ (for a comprehensive review see^19,20^). Currently, these studies do not offer a converging answer as to which neural patterns underly word-processing, which is why existing data can be interpreted in the context of either the dual coding or context availability theory^7^. These inconsistencies can be attributed to many factors, including the variation in task or stimulus material^17^ and the limited temporal resolution of tools such as fMRI and PET which fall short in capturing the temporal development during word comprehension.

Electrophysiological tools such EEG and MEG ((M)EEG), by contrast, provide more insight into the temporal dynamics of the electrophysiological processes that underlie word processing. Even though this temporal accuracy comes at the expense of a lower spatial precision compared to neuroimaging techniques such as fMRI, source reconstruction techniques in (M)EEG are continually improving their spatial resolutions^21^. In search for the concreteness effect, EEG studies have successfully shown a decrease of a negative amplitude deflection for concrete words around 400 ms post stimulus (the time window associated with semantic processing)^9,22,23^ which indicates a facilitated semantic processing of these words. This deflection is assumed to be responsible for word encoding and retrieval, and is called the N400 component^2,24,25^. Retrieval of context details in concrete words is related to a later, positive amplitude (P600-like component). Due to greater retrieval of contextual information at distinct temporal stages, these results could explain the advantage of concrete over abstract words^26^. Similar to fMRI/PET studies, those relying on (M)EEGs have been inconclusive in their support or rejection of current theories. For example, studies have attributed the observation that N400 amplitudes for concrete words are smaller over the right but not the left hemisphere to the dual coding theory, as they assume that this reflects an increased facilitation in the nonverbal system of the right hemisphere^9,27–29^. In another study, an increased MEG response in the left frontotemporal areas for concrete and in the right anterior temporal areas for abstract words has been observed during the stage of semantic processing. While this appears to diverge from the dual coding pattern, the authors still consider their results to concur with this theory, as they assume that decreased MEG activity suggests a more “efficient” representation of these word types in the left and right hemispheres^30^. Other studies seem to favor the context availability theory. For example, using high-density EEG and source localization, abstract words have been shown to have a larger area of activation compared to concrete words in areas limited to the left hemisphere^2^. An overall larger activation of abstract words under certain task conditions has also been observed^31^ and has been shown to increase even more during the stage of semantic processing^32^.

In summary, the inconclusiveness of the current evidence indicates that there is a clear need to fill the gap between the hypotheses of language processing and the neuroscience behind it^33^. More recent theories have already tried to emphasize the grounding of abstract words in either the same sensory and motor systems as for concrete words^34^, in emotion^35,36^ or in social systems^8^. However, given the complexity and controversial nature of the issue as well as the theoretical shortcomings, our tools have been too limited conclusively to support or reject any theory of word processing. The above-mentioned controversy would suggest that there is a need for a clearer method to investigate the differences between the processing of concrete and abstract words. It is worth establishing a more complete picture of not only the temporal, spatial and spectral dynamics of brain areas, but also of the nature of the interactions between them. It would therefore be beneficial to conduct a connectivity analysis of high spatio-temporal resolution data, such as that generated by EEG, to understand the dynamic interactions that support language processing^37,38^.

In general, however, little is mentioned about interactions between brain regions during word comprehension. This lack of knowledge can lead to a misinterpretation of the obtained results, also in view of the lingering ambivalence. In the last decade, the scientific community has been increasingly investigating brain connectivity for a better understanding of cognitive processes^39,40^. Indeed, since cognitive functions rely on connectivity within large-scale networks^41^, approaching cognitive processes from the point of view of connectivity can resolve fundamental questions in language processing, such as whether word processing is modular or interactive, parallel or serial^37^. This can then also help distinguish between feedforward and feedback processes in language^42^. For example, study^43^ showed that different frequency bands are involved in the bottom up and top down processing of natural reading states. Other studies have used symmetrical connectivity measures to analyze linguistically complex words and have found a left-lateralized frontotemporal cross-cortical interaction^44^.

By investigating the connectivity between different brain areas during the processing of concrete and abstract words, it is possible to improve the current neurobiological models for these observations. So far, only a few experimental studies have investigated the functional connectivity for abstract and concrete word processing. For instance, when testing the imaginability of characteristics of auditorily-presented animals, the authors of study^45^ found higher engagement of the linguistic and visual regions in the right hemisphere when imagining concrete (e.g. long ears of a rabbit) compared to abstract characteristics (e.g. braveness of a lion). According to the authors, this result supports the dual coding theory. Additionally, a stronger connectivity pattern for concrete characteristics was found in both the left and right hemispheres. However, since the study tested imagery rather than an actual reading process, the question remains as to what happens in the brain when abstract or concrete words are read. Furthermore, as the study was done with fMRI, the authors could not describe the spectral and temporal characteristics of the identified networks. State-of-the-art high-density EEG systems provide a spatial resolution sufficient for examining the brain areas involved in word processing^25,32^. To the best of our knowledge, there is only one (M)EEG connectivity study that compared brain connectivity when processing concrete and abstract words^46^. Using combined EEG and MEG with an explicit concreteness task, the authors observed significant differences in connectivity between the left anterior temporal lobe and the angular gyrus. The issue in this study is that the explicit task might influence the results since, in natural settings, we do not judge particular characteristics (in this case concreteness) of a word that is read. Hence, it is still not clear whether the brain would respond differentially to abstract and concrete words in more natural conditions and without imposing a task.

In our study, we investigated the spatio-temporal dynamics of visual word processing both in terms of neural activation, frequency and directionality of information flow. We additionally determined how concreteness of a word affects these processes. To do so, we adopted a comprehensive approach. For optimal spatial resolution, we opted for a high-density EEG setup^32^. In EEG connectivity studies, spurious connectivity can occur due to the spatial spread (resulting from volume conduction) during which signals coming from different neural sources are mixed before reaching the scalp surface. Thus, connectivity measured on this surface could reveal artificial or spurious connections which do not result from true neuronal interactions^47^. Source localization attempts to “unmix” the recordings to arrive at the location and the activation patterns of the underlying neural source. To capture true connectivity, we thus conducted our analysis at the source rather than at scalp level^48^. We defined our brain regions of interest (ROI) empirically to allow the inclusion of ROIs not predicted by current theories^37^. We also selected ROIs based on two distinct measures of neural activation in order both to distinguish between differences in connectivity and differences in activation, and to identify common and differential activation between abstract and concrete word comprehension.

For our connectivity measure, we adopted a method based on the concept of Granger Causality (GC)^49,50^ called partial directed coherence (PDC)^51^. Note that Granger Causality should not be confused with causality in the conceptual sense of “A brings about B”. Rather, it is an estimation of causal statistical influences without the need for a physical intervention^52,53^. Functional connectivity measures such as GC belong to a branch of popular connectivity methods which include information on the directedness of information flow^47^ enabling scientists to estimate the temporal precedence of the influence of one variable in a system on another^52,53^. Additionally, as GC is a data-driven approach it does not assume predefined connections between variables (in our case ROIs). This renders GC particularly attractive for our purposes. Apart from a few attempts^54,55^, the spatio-temporal patterns of interactions between brain areas on word processing has yet to be defined. Our method would thus enable us to address the issue of defining the spatio-temporal pattern without limiting ourselves to a prior definition of the ROIs. In the traditional definition of GC, connectivity is defined in the time domain and only between two variables. However, the PDC method accounts for multiple brain areas (i.e. multivariate case)^51^, meaning that we can satisfy the requirement of GC to take all ROIs affecting the system into account^49^. Moreover, the PDC is computed in the frequency domain enabling us to find distinct patterns of connectivity in different frequency bands. Furthermore, we adopted an extended version of PDC that is time-varying and multi-trial, both characteristics suited for this study. The former because in cognitive processes like word comprehension, functional brain networks change on a sub-second temporal scale^56^. In order to capture these transient alterations in connection strength, time-varying versions of the PDC algorithm have been developed^57,58^. The latter because our algorithm is trained on multiple trials (i.e. multi-trial approach). As our study requires the collection of multiple trials per word type, models are needed that can account for the trial-to-trial variability^59,60^. As far as we know, the current state-of-the-art method of connectivity has not yet been applied to understand the patterns of word processing. In this study, we thus attempt to investigate the dynamic and directional connectivity patterns elicited during implicit processing of abstractness when reading single words.

## Materials and Methods

### Participants

Twenty-four healthy native Dutch speaking participants (13 males, 11 females, average age 22 ± 4 years, all right handed, paid) participated in our study which was approved by the Ethics Committee of the Leuven University Hospital and conducted according to the latest version of the Declaration of Helsinki. All recruited subjects were given instructions regarding the task at hand and were informed about data collection and information privacy regulations. They were invited to read and sign the informed consent form. No participant reported any history of neurological or psychiatric disorders. All participants had normal or corrected-to-normal vision.

### Materials

For our task, we chose a categorization paradigm in which participants were asked to decide whether or not the presented word belonged to the semantic category of “colors.” The categorization task ensured that our subjects were attentive and involved in semantic processing and not merely in lexical access (as would be the case with a lexical decision task). At the same time, this task, devised to distract participants, kept the subject unaware of the purpose of the experiment and therefore enabled a more natural setting for word comprehension. However, the color category itself served as a “filler” category with words that were colors discarded from further analysis (see experimental set-up). For the words of interest, we presented Dutch abstract and concrete nouns taken from the database with concreteness ratings of 30,000 Dutch words^61^. Abstract words were selected with a concreteness rating of maximum 2.5 and concrete words with a minimum of 3.5 (on a scale from 1 to 5). This choice ensured that words on both sides of the concreteness spectrum are selected and that there is a high statistical difference between the concreteness rating of the two groups. Based on a t-test, concreteness ratings of abstract and concrete words were found to be statistically different [p < 0.1e-10, mean and standard deviation (in brackets) for concrete and abstract words were respectively 4.33/1.97 (0.43/0.28)].

Previous research has shown that abstract words tend to be more emotionally charged than concrete words^35,62^. Therefore, unlike previous studies^30,46^, we controlled our stimuli for the three dimensions of affective meaning, also known as Osgood values. These are three independent dimensions marked by the following polar adjectives: “valence” (positive vs negative), “potency” (strong vs weak) and “activity” (active vs passive). Definitions of the individual stimulus groups and an example per group can be found in Table 1. These dimensions have been shown to quantify the affective meaning of verbal terms^63^ and to reveal a differential neural pattern of activation^32,64^. Ratings for Osgood values were obtained from database^65^ of 4,300 words with norms of valence, arousal and dominance. To be included in the stimulus list, words along each dimension needed to have a rating of a minimum of 4 or a maximum of 3 on a 7-point Likert scale [mean and standard deviation (in brackets) for concrete and abstract categories were 4.19/4.03 (1.04/1.03) for arousal, 3.74/3.74 (1.15/1.48) for potency and 4.23/4.06 (0.81/1.03) for valence]. Additionally, word length (number of letters) and word frequency for both word types were controlled using the Dutch CLEARPOND software (word length mean = 7.7, std = 1.4 for concrete and mean = 8.5, std = 2.3 for abstracts; word frequency mean = 13.7, std = 34 for concretes and mean = 17.8, std = 60.3 for abstracts)^66^. These restrictions on word selection resulted in a total of 50 words per category group, except for the concrete passive and concrete weak group for which we could only retrieve 25 words in the database for each group. Furthermore, words were pseudo-randomly organized in such a way that no two consecutive words would have a forward association strength higher than 1, where forward association strength was taken from the Dutch free association network created by De Deyne & Storms^67^. A complete list of the words of interest presented during the task is given in supplementary material section F.

**Table 1:** Examples of trials. All trials were presented in the participant’s native language (Dutch). Fillers constitute names of colors (not shown in the table).

Trial Type	Abstract	Concrete
*Active*	ambitie (ambition)	vloedgolf (tidal wave)
*Passive*	pensioen (pension)	tapijt (carpet)
*Positive*	beroemd (famous)	speelgoed (toy)
*Negative*	vloek (curse)	vulkaan (vulcano)
*Weak*	armoede (poverty)	kalkoen (turkey)
*Strong*	competitief (competitive)	spinazie (spinach)

English translation (between brackets) is for illustrative purposes only.

### Experimental set-up

Participants were tested in a sound-attenuated, darkened room with a constant temperature of 20 degrees, sitting in front of an LCD screen at a distance of about 70 cm. In accordance with the international extended 10–20 system, EEG was recorded using 128 active Ag/AgCl electrodes (SynampsRT, Compumedics, France). Two of these electrodes served as ground (AFz) and reference (FCz). The EEG signal was recorded at a 2 KHz sampling rate. All electrodes were mounted on an electrode cap placed on the subject’s head (Easycap, Germany).

During individual trials a single white word was shown in the middle of a black screen for 300 ms followed by a question mark lasting for 1 second. Prior to each trial a fixation cross appeared on the screen for 700 ms cueing subjects to fixate their gaze on the middle of the screen. Only when the presented word was a color (i.e. a categorization paradigm), participants were asked to press the mouse button at the moment they saw the question mark. After that, participants received the visual feedback “kleur!” (color) when the button was pressed correctly and “fout!” (wrong) otherwise. Since words of the category *color* were not relevant to the task and required a motor response that could interfere with the electrophysiological response^68^, we removed these trials from our analysis. Therefore, we did not control for the characteristics of these (filler) words.

### EEG signal pre-processing

EEG recordings from three participants were lost due to technical issues. For the remaining 21 participants, we re-referenced their EEG recordings offline from the original central reference to a linked mastoid reference. Eye movements were corrected using the method described in^69^. The recordings were then filtered with the help of a 4^th^ order Butterworth filter in the range of 0.5–30 Hz to discard low and high frequency noises. To reduce the computational effort, data were down-sampled to 200 Hz. These parameter settings for multi-trial ERP data have been validated previously^70,71^. The recordings were epoched with a window between 200 ms pre- and 1000 ms post-stimulus intervals and baselined with the 200 ms pre-stimulus time-window. Based on visual inspection, recordings from bad channels of each subject were removed (36 ± 16 channels out of 128 per subject on average). Of the remaining channels, we then eliminated all trials whose maximum EEG amplitudes exceeded ±150 µV. On average, this eliminated about 17% of the trials per subject. As multivariate causality measures are generally sensitive to data pre-processing and are recommended only insofar as necessary to eliminate noise^72^, we opted to eliminate trials rather than apply an additional artefact reduction step. In total, we had 556 ± 132 trials remaining per subject. For abstract and concrete words, the total comes to respectively 251 ± 59 and 212 ± 47 trials per subject.

### Scalp analysis

Scalp analysis was conducted with the same methods as described in^32^, where a mass-univariate approach was adopted by means of a linear mixed effect model. In the current study, subjects were regarded as random, and abstract and concrete categories as fixed effects. The dependent variable was the EEG amplitude, averaged over 50 ms time bins between 0 and 700 ms. We corrected for multiple comparisons over the electrodes using cluster-based inference by taking a cluster size of 10 adjacent electrodes. Additionally, only clusters that were statistically significant for at least two consecutive time bins were considered. For a more detailed explanation on this method and its advantages see^32^.

### Source Localization and ROI selection

#### Source localization

We conducted source localization using the Brainstorm toolbox^73^. For our head model, we took the ICBM-152 template and for the forward model OpenMEEG BEM^74^, where the source space was restricted to the cortical surface which we divided into 15,000 dipoles. The noise covariance matrix was obtained by merging the matrices calculated from the baseline of all trials. We used source data to estimate the current density maps based on the minimum-norm estimate (MNE). These are the estimated time courses necessary for Granger causality analysis (see next section). However, prior to our connectivity analysis, we identified our regions of interest (ROIs) across the cerebral cortex.

#### ROI selection

ROI selection for connectivity analysis is a complicated and relatively neglected issue. Since it is a requirement to have all potentially causal signals included in the Granger causality analysis, one could be tempted to include the entire cortical surface. However, this is unrealistic since the computational complexity of the multivariate Granger causality quickly increases as *O*(*M*^2^*p*), where *M* denotes the number of variables and *p* the order of the model. Including many time series increases redundancy which could demote model sensitivity^37^. Redundancy is inherent in the spatially smooth solutions of source distributed algorithms used for source localization. This leakage between brain sources makes it difficult to distinguish true from spurious connections^75^. For this reason, we selected a few anatomically well-separated ROIs based on measures of neural activation (see below), which is one of the more common approaches^37,76^. The advantage of our data-driven method is that it can unveil ROIs when there is little prior information on which ones to choose. This is more in line with the exploratory nature of Granger causality. However, the downside is that our method could be blind to ROIs that play a causal role in neural dynamics but that are downregulated for the considered subject task^37^.

To find differences in the patterns of connectivity between abstract and concrete words, we were interested in seeing whether differences in connectivity would concur with a difference in activation or whether connectivity patterns can differ even when activation patterns are similar. For this reason, we identified ROIs based on two measures of activation, one to identify neural activity that is common between both paradigms (method A) and a second to identify differential activation between abstract and concrete words (method B).

A. For ROI selection, we adopted an approach similar to that of^30^ where a noise-normalized version of MNE, called dynamic statistical parametric mapping (dSPM)^77^, was constructed for all collective trials (“collective activation”). The dSPM technique yields an assessment of the signal-to-noise ratio of the current estimate. ROIs are then selected based on a careful examination of the map using anatomical landmarks, whereby we refrained from including deep cortical areas as the contribution of their activity to scalp EEG is still very controversial^30,78^. Additionally, time series originating from nearby areas were inspected to ensure that they were not redundant.
B. To identify ROIs showing differential activity between abstract and concrete words, we adopted the mass-univariate approach with cluster-based correction for multiple comparisons. This method is described in^32^, but for the sake of clarity we will briefly outline it here too. A mass-univariate approach using a linear mixed effect model was employed in^79^ with subjects taken as random and semantic abstractness as fixed effects. The dependent variable was the dipole amplitude, averaged over 50 ms time bins between 0 and 700 ms. Averaging was performed for each of the 15,000 dipoles. Test results with p-values below 0.05 were considered significant. Furthermore, we corrected for multiple comparisons using the cluster-based inference adapted from random field theory^80^, i.e. we only considered regions statistically significant for a minimum of two consecutive time windows over a connected cortical region with a minimum cluster size of 3 cm^2^.

The results of this analysis are shown in Table 2, where it can be seen that method A resulted in the 10 ROIs shown and method B was additionally able to replicate ROI 7 and 8. In both cases, within each anatomically well-separated ROI, we selected one representative time course by taking that with the highest power among all time courses in the ROI. This method has been shown better to capture the dynamics and phase of the signal which would otherwise become lost when averaging an already smooth distribution of sources^78,81^. To confirm that this chosen time course is a good representation of the ROI, we manually inspected our ROIs to ensure that they were small enough to have similar activation time courses throughout the region. Furthermore, some ROIs spanned both sulci and gyri, causing the orientation of the dipoles to be opposite within an ROI. This however, does not pose a problem for the Granger Causality analysis. GC is invariant under rescaling of variables in the system and therefore only *independent* information of the past of one variable improves the prediction of another^53^. An example of the time series within an ROI is shown in supplementary material section A.

**Table 2:** Anatomical label and MNI coordinates of vertices with 10 ROIs whose time courses were used for subsequent analysis. All ROIs were identified using method A.

ROI	MNI coordinates	Time window of difference	P value (corrected)	Cohen’s d	Size (cm^2^)	Polarity	Stronger Condition
1 right superior occipital gyrus	[35.6, −55.6, 54.4]	n.a	n.a	n.a	49.21	n.a	n.a
2 left orbitofrontal gyrus	[−47.4, 45.6, −18.9]	n.a	n.a	n.a	35.64	n.a	n.a
3 left anterior temporal lobe	[−48.6, 11.9, −43.9]	n.a	n.a	n.a	32.38	n.a	n.a
4 right anterior temporal lobe	[48.9, 13.5, −43.3]	n.a	n.a	n.a	12.51	n.a	n.a
5 left posterior middle temporal gyrus	[−60.3, −68.4, 0.8]	n.a	n.a	n.a	17.35	n.a	n.a
6 right middle frontal gyrus	[37.3, 9.6, 64.2]	n.a	n.a	n.a	27.14	n.a	n.a
7 left inferior temporal gyrus	[−62.5, 25.6, −31.6]	300–650 ms	0.0225	0.022	35.23	Ab > Co	|Ab| > |Co|
8 bihemispheric superior parietal lobule	[13.0, −47.7, 79.8]	550–750 ms	0.0167	0.014	9.79	Ab > Co	|Ab| < |Co|
9 right middle and superior temporal gyri	[71.4, −22.3, −6.7]	n.a	n.a	n.a	20.41	n.a	n.a
10 right orbitofrontal gyrus	[36.3, 33.7, −22.6]	n.a	n.a	n.a	34.65	n.a	n.a

Regions 7 and 8 were identified by using both A and B. For method B, characteristics of the differential activity (time window, p value, effect size, polarity and strength of activation) are given which are not applicable (n.a.) for method A. Ab: Abstract, Co: Concrete.

### Granger causality

In this study, we adopted a multivariate time-varying directed connectivity approach based on Granger causality, called partial directed coherence. The time-varying multivariate process is defined as follows:

1 $$y(n)={\sum }_{p}^{p}{A}_{k}(n)y(n-k)+e(n)\,$$

where $y\in {R}^{m\times N\times T}$ represents the multi-variate (*m*), multi-trial (*T*) timeseries of length *N*, and *n* the *n*-th time bin of the *N* samples in each trial. The order of the model is *p*, which determines the maximum number of delayed observations in the model. For example, since we were using a sampling rate of 200 Hz, a model order of 10 took the past 50 ms of the timeseries into account. *A*_*k*_(*n*) represents the time-varying autoregressive parameters which we estimated by using a multi-trial adaptation of the General Linear Kalman filter. This has been shown to outperform other methods, including the recursive least squared method^57,59,81^. In the Kalman filter, autoregressive parameters are estimated using a linear state-space model, allowing for non-stationarity. This is a highly sought-out quality in our study, since EEG is essentially non-stationary^82^.

In the case of the General Linear Kalman Filter (GLKF) model, two parameters needed to be defined: the first was the model order which is the maximum number of lagged observations. The second was the adaptation coefficient which is common in recursive algorithms and regulates the adaptation speed of parameter estimation. Parameter estimation was crucial to reach a good estimation of connectivity. Indeed, a low model order can lead to a poor representation of the data, whereas a high order can lead to incorrectly rejecting the null hypothesis (type I error). To tune these parameters and validate the model, we took several steps as described in supplementary material section B.

Once the model order was established and the autoregressive parameters estimated, we inferred Granger-based connectivity from it. In the multivariate case, the Fourier transform of the multivariate time-varying autoregressive parameters was computed to obtain the spectral density matrix $(f,n)\in {R}^{m\times m\times f\times n}$:

2 $$A(f,n)={\sum }_{k=1}^{p}{A}_{k}(n){e}^{-i2\pi fk},\,\bar{A}(f,n)=I-A(f,n)$$

Given the spectral density matrix, several connectivity metrics can be defined. We opted with the partial directed coherence (PDC), a spectral measure of directed information flow for a multivariate autoregressive (MAR) model based on the more fundamental concept of Granger Causality. This measure can distinguish between direct and indirect connections correctly identifying interactions even in relatively noisy data:

3 $${\pi }_{ij}(f,n)=\frac{{\bar{A}}_{ij}(f,n)}{\sqrt{{\sum }_{r=1}^{m}{\bar{A}}_{ir}(f,n){\bar{A}}_{ir}^{H}(f,n)}}$$

where the following normalization properties hold:

4 $$0 < {\pi }_{ij}(f,n) < 1\,{\rm{and}}\,{\sum }_{i=1}^{N}{|{\pi }_{ij}(f,n)|}^{2} < 1\,{\rm{for}}\,{\rm{all}}\,1\le j\le N$$

Note that, in the current definition of PDC, every connection *π*_*ij*_ is the information flow from region j to i and is normalized by the strength of all incoming connections to the *i*-region, which produces stable and interpretable results^51,83^.

### Statistical analysis of Granger connectivity

All implementations of model estimation, validation and statistical analysis were done using custom-written scripts or scripts modified from available MATLAB packages such as the MVGC toolbox^84^, the SIFT toolbox^85^, the WOSSPA package^86^ and the ARfit package.

#### Testing for significant non-zero information flow

The multi-trial GLKF algorithm was trained on all trials for each subject simultaneously and presented as a single time-frequency plot per subject. To test for statistically significant non-zero connections, we first calculated PDC values on grouped subjects of abstract and concrete words per subject. From the same dataset, we generated our null distribution by randomizing the phase of each trial while preserving the amplitude distribution^87^. Phase randomization was implemented by applying a fast-Fourier transform (FFT) to obtain the complex power spectrum, replacing the phases with those of a uniform random matrix. Then, to obtain the surrogate data we applied the inverse FFT (implemented using the SIFT toolbox^85^). Since information flow critically depends on phase information, the estimated connectivity observed should be statistically larger than the connectivity estimated from the surrogate data. The latter, in fact, represents the behavior of the null hypothesis case. The effectiveness of this technique has been validated on simulation examples^88^. In this way, we could conservatively discard a substantial set of connections for which the estimated Granger causality was likely to result from spurious connectivity due to volume conduction^43^. From the null distribution, we calculated once more the surrogate PDC values to test for significance using a one-sided within subject cluster-based permutation test as described in^89^. Clusters were defined as datapoints adjacent in time and frequency. The significance of all clusters was calculated under the permutation distribution of the maximum cluster statistics so as to control the family-wise error rate (FWER) for all clusters. The cluster-based permutation test was performed between all ROIs resulting in a total of 100 cluster-based permutation tests in which we corrected for multiple comparisons (10 ROIs resulted in 100 comparisons) using Bonferroni correction of the critical alpha level (α = 0.0005). The test statistics were obtained on 10000 random partitions. In addition to the level of significance, we reported the effect size (Cohen’s d) between the surrogate and real PDC values, which was estimated as an indication of the strength of the connection.

#### Testing for significant differences in information flow between abstract and concrete trials

Once significant non-zero information flow had been determined with the help of the cluster-based permutation method described above, we tested for differences between information flow of abstract and concrete words using a paired sample t-test. In this case, we only analyzed the connections that were statistically significant in the cluster-based permutation test with the effect size in the “very large or huge” range (see section results: Granger Causality, experimental data). We believe these connections to be the most robust and strongest, which is why we focused our analysis on them. For simplicity, we also excluded edges showing this range of effect size for only abstract or concrete words. For visualization purposes, an alternative representation of the same results is also shown as a bar plot where an averaged sum of the incoming and outgoing connection strengths is represented (see section Results: Granger Causality, experimental data). We limited our statistical analysis to comparing connectivity only during the time-span where significant differences were observed in amplitude using method B in ROI identification (see section results: source localization and ROI selection). In order to reduce the number of pairs included in the multiple comparison analysis, we analyzed only the time-span where a significant difference in neural activation was observed. Within this time-span, we averaged over 100 ms time bins with 50 ms overlap. Furthermore, we only considered results that were significant for a frequency range of at least 3 Hz. An exploratory analysis of these connections for the whole timespan is reported in supplementary material section D.

#### Consistency of PDC

An important question is how consistent our PDCs are. Since the entire set of available trials is used to estimate Granger causality per subject and condition, there is no estimate of its variability. To accommodate this, we performed bootstrap resampling per subject and condition, and generated 100 resampled datasets, i.e. we repeated the exact same analysis procedure as we did with our original dataset but randomly resampled by replacing the trials within each condition and subject. The size of the resampling dataset was the same as in the original analysis. From this, we calculated the average connectivity over all subjects per bootstrap resampling. The standard deviation over all connectivity pairs in time-frequency showed a maximum of 0.05 (5% of the maximum estimate). A z-score analysis of all bootstrap trial showed that 95.91% of the estimates lie within 2 standard deviations of the mean for each estimate. A further detailed analysis of the inter-subject variability for different connection pairs can be found in supplementary material section E.

In addition to the above-mentioned analysis, we were also curious to see how this variability would differ if a sample smaller than the whole dataset were used. As mentioned in the pre-processing section, we had an average of 550 trials per subject for both conditions, meaning that around 270 trials per condition were used to estimate the autoregressive parameters. We repeated the bootstrap analysis for 15, 25, …, to 95% of all available trials to estimate the variability. Results show a slow decline in standard deviation from 0.09 to 0.08 up until 85% of the trials are used, after which the standard deviation drops from 0.08 to 0.05, ultimately reaching 0.03 at 95%. As 85% of trials corresponds to ~230 trials, we propose that this be the minimum amount of trials required for a reliable estimation of the autoregressive parameters in our case.

## Results

### Scalp results

**Figure 1:**
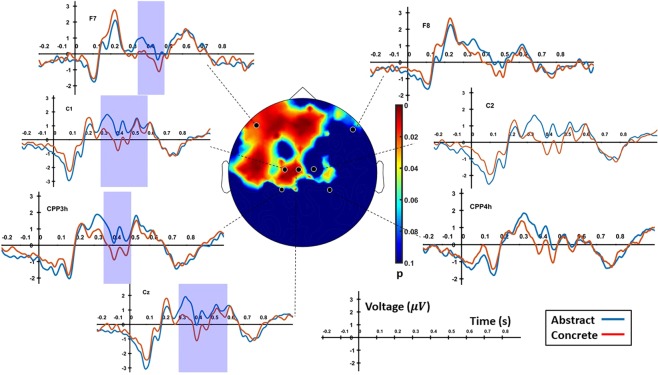
Scalp EEG plots for abstract and concrete word trials for seven representative electrodes (F7, F8, C1, C2, CCP3h, CCP4h, and Cz).

An initial analysis of the scalp electrodes already revealed some details of the differences between abstract and concrete words. Linear mixed effect analysis on scalp electrodes with subject as random effect and cluster-based correction for multiple comparisons (only effects were taken into account that lasted for at least 100 ms over several adjacent electrodes) showed a left-lateralized difference between the two conditions during the 300–500 ms time window (p = 0.0085). Figure 1 shows a map of the scalp regions significant during this time-window together with the time series of some representative channels for the mentioned effect. Some differences can already be noticed at 100 ms, where a more negative deflection is observed for abstract words in most electrodes. However, a statistical significance for the difference is only reached from 300 ms onwards. This significance lasts until 600 ms in the left centroparietal electrodes like C1 and Cz, and until 500 ms in left frontal electrodes like F7. In this time window, the concreteness effect is expressed by a more positive amplitude for abstract words during this time window compared to concrete words. Similar to^9^, differences between word types are greater over anterior than over posterior sites (average Cohen’s d effect size between 300–500 ms 0.0318 of anterior versus 0.0022 for posterior sites). In that study^9^, however, the differences were greater over the right compared to the left hemisphere, which could be due to the implicit task of word categorization.

### Source localization and ROI selection

**Figure 2:**
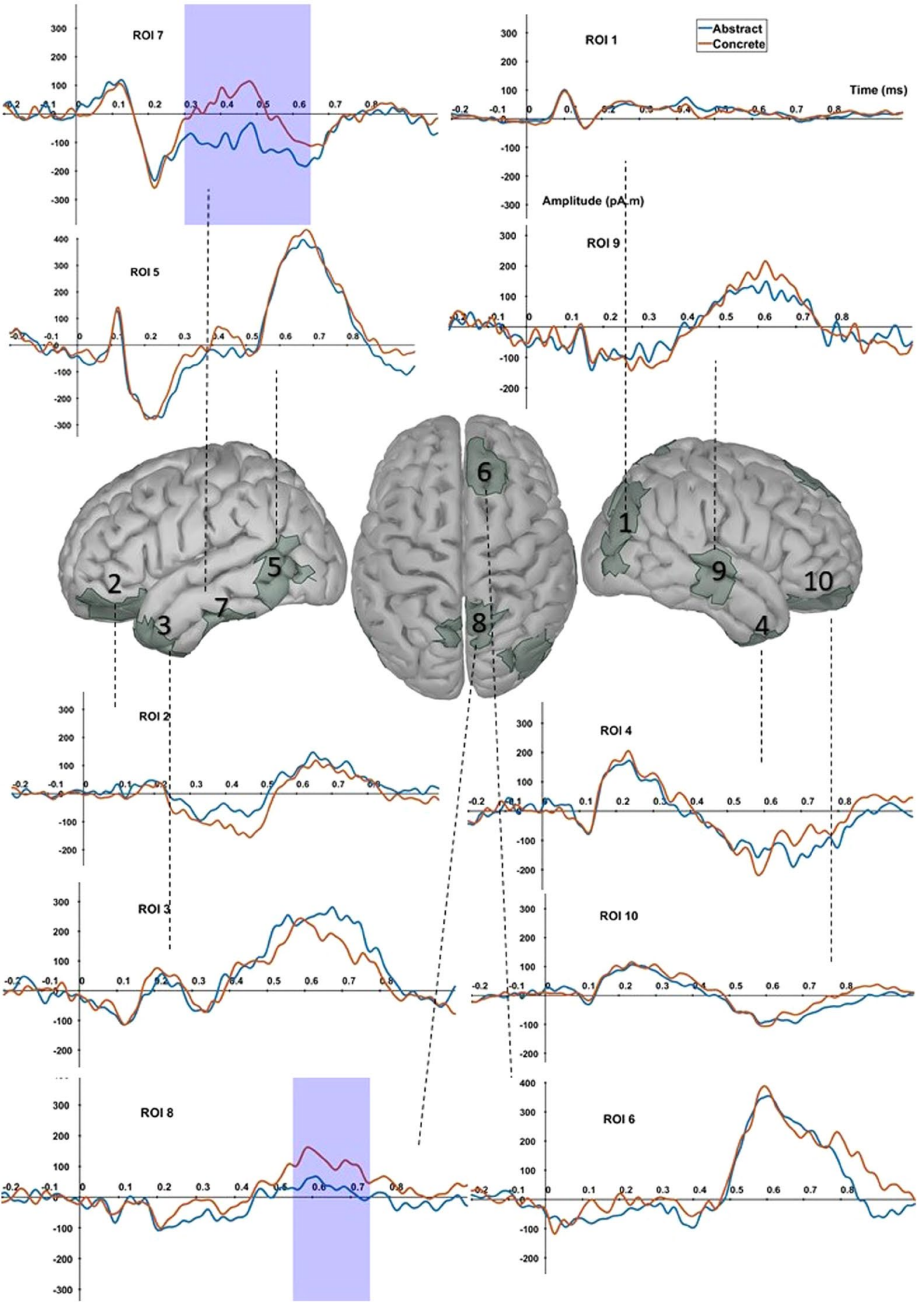
ROIs selected for connectivity analysis. All areas have been found by means of method A, which shows common activity between abstract and concrete trials. Regions 7 and 8 have been re-identified using method B, i.e. they represent regions with statistically significant differences between abstract and concrete trials obtained by a mass-univariate linear mixed effect model with cluster-based correction.

Having found confirmation in the scalp analysis that there is indeed a difference between abstract and concrete words, we performed source space analysis to obtain the ROIs that would be taken into our model of connectivity analysis in later steps. Table 2 shows the anatomical label and Montreal Neurological Institute (MNI) coordinates of ROIs, with the grand average time courses shown in Fig. 2 (Note that columns “polarity” and “stronger condition” are only defined in method B, respectively representing the relation for the dipole amplitude and the strength of the rectified amplitudes^32^). When searching for patterns of common activity using method A (see Materials and Methods, section on Source localization and ROI selection), 10 ROIs were identified. These ROIs represent areas where collective activity of both conditions exhibited a significant source signal to noise ratio^30^. Very interesting to note is that when searching for differential patterns of activation (method B), ROIs 7 and 8 (left inferior temporal gyrus and bihemispheric superior parietal lobule) were re-identified. This shows that despite a statistically significant difference in amplitudes, the combined activity of both conditions was high enough for these ROIs to exhibit a strong power compared to the baseline activity. Using the methods as described in the methods section, we endorsed – at least partially – the regions hypothesized by Lau *et al*.’s model of semantic processing^54^.

Furthermore, Fig. 2 also shows the respective time course for each of these ROIs which were obtained by taking the dipole with the highest power for each region. Similar to the results of the scalp analysis, a significant difference between abstract and concrete words starts at 300 ms. This difference is localized at the left inferior temporal gyrus. Additionally, a statistical difference can be observed in the superior parietal lobule of both hemispheres at a slightly later time window. For other ROIs, none of these differences reached statistical significance even though some differences can be seen, such as in the case of the right anterior temporal lobe starting at 600 ms.

### Granger Causality

#### Simulated data

After having selected our ROIs, we first validated our time-varying, multivariate and multi-trial connectivity method using a ground truth, i.e. simulated data. The simulation example was taken from^90^, a multivariate autoregressive model of order 2 with 3 variables, *x*_1_, *x*_2_, *x*_3_. The method we used is largely similar to that of^90^ except that in our case the PDC technique was adapted for multiple trials of the same phenomenon, whereas in^90^ it was applied to continuous data:

$${x}_{1}(n)=0.5{x}_{1}(n-1)-0.7{x}_{1}(n-2)+{c}_{12}(n){x}_{2}(n-1)+{w}_{1}(n)$$

$${x}_{2}(n)=0.7{x}_{2}(n-1)-0.5{x}_{2}(n-2)+0.2{x}_{1}(n-1)+{c}_{23}(n){x}_{3}(n-1)+{w}_{2}(n)$$

$${x}_{3}(n)=0.8{x}_{3}(n-1)+{w}_{3}(n)$$

where

$${c}_{12}(n)=\{\begin{array}{cc}\frac{n}{L} & n\le L/2\\ \frac{(L-n)}{L} & n > L/2\end{array}\,{\rm{and}}\,{c}_{23}(n)=\{\begin{array}{cc}0.4 & n\le 0.7L\\ 0 & n > 0.7L\end{array}$$

**Figure 3:**
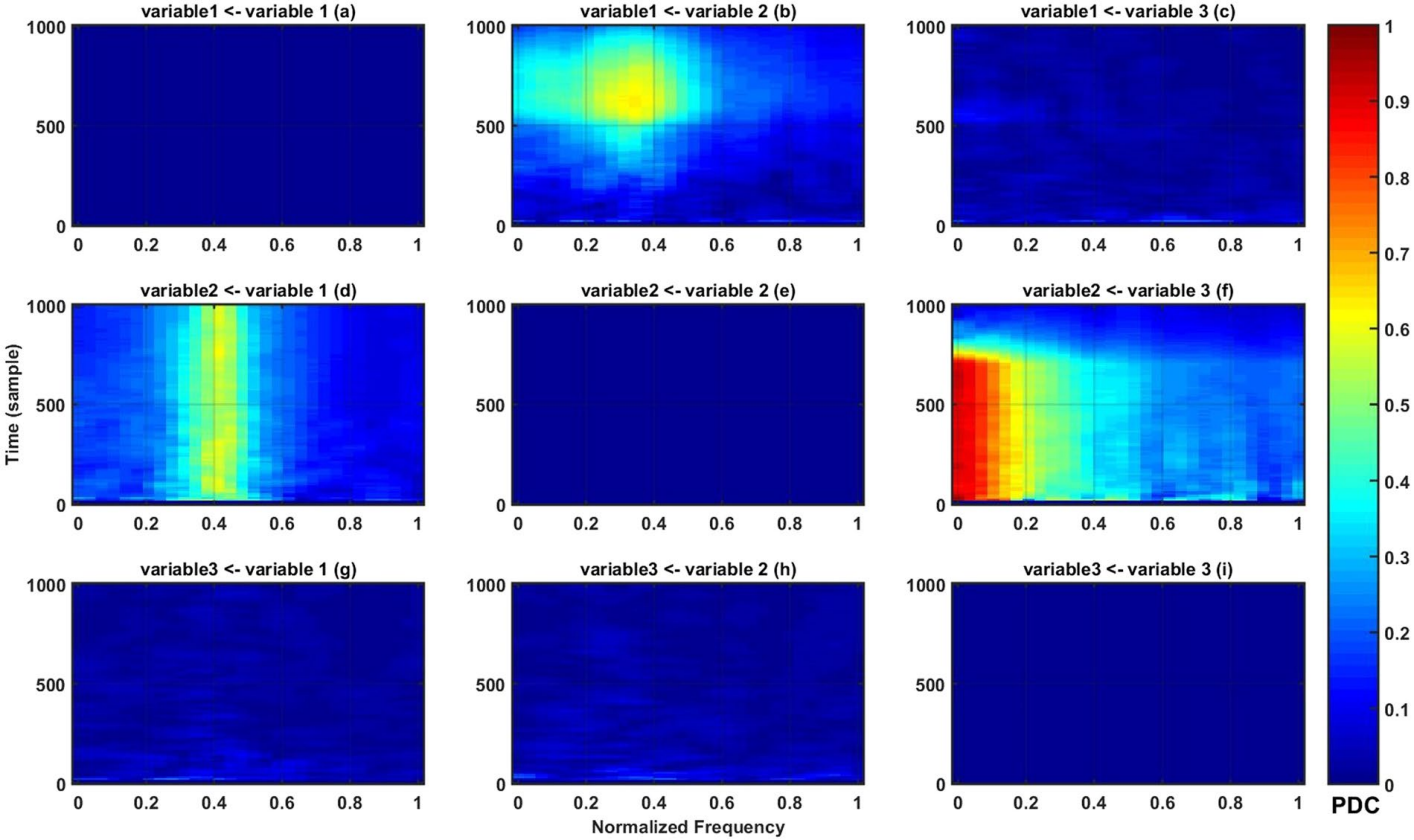
Multivariate Granger causality of simulated data plotted in terms of partial directed coherence (PDC) (cf. color scale) in the time-frequency domain.

Trial length *L* is equal to 1000 data samples per trial of which we generated 100 trials. Figure 3 illustrates our PDC estimates using the multi-trial general linear Kalman filter for parameter estimation. The figure features 3 × 3 time-frequency windows each of which displays information flow between two variables. Some patterns of information flow change with time. This can, for example, be seen in window (b), where a flow of information from variable 2 to 1 appears after passing half the time window (variable *c*_12_(*n*) varies with time). These simulations confirmed the validity of our method in capturing the varying nature of the connectivity pattern for the next stage of our analysis.

#### Experimental data

**Figure 4:**
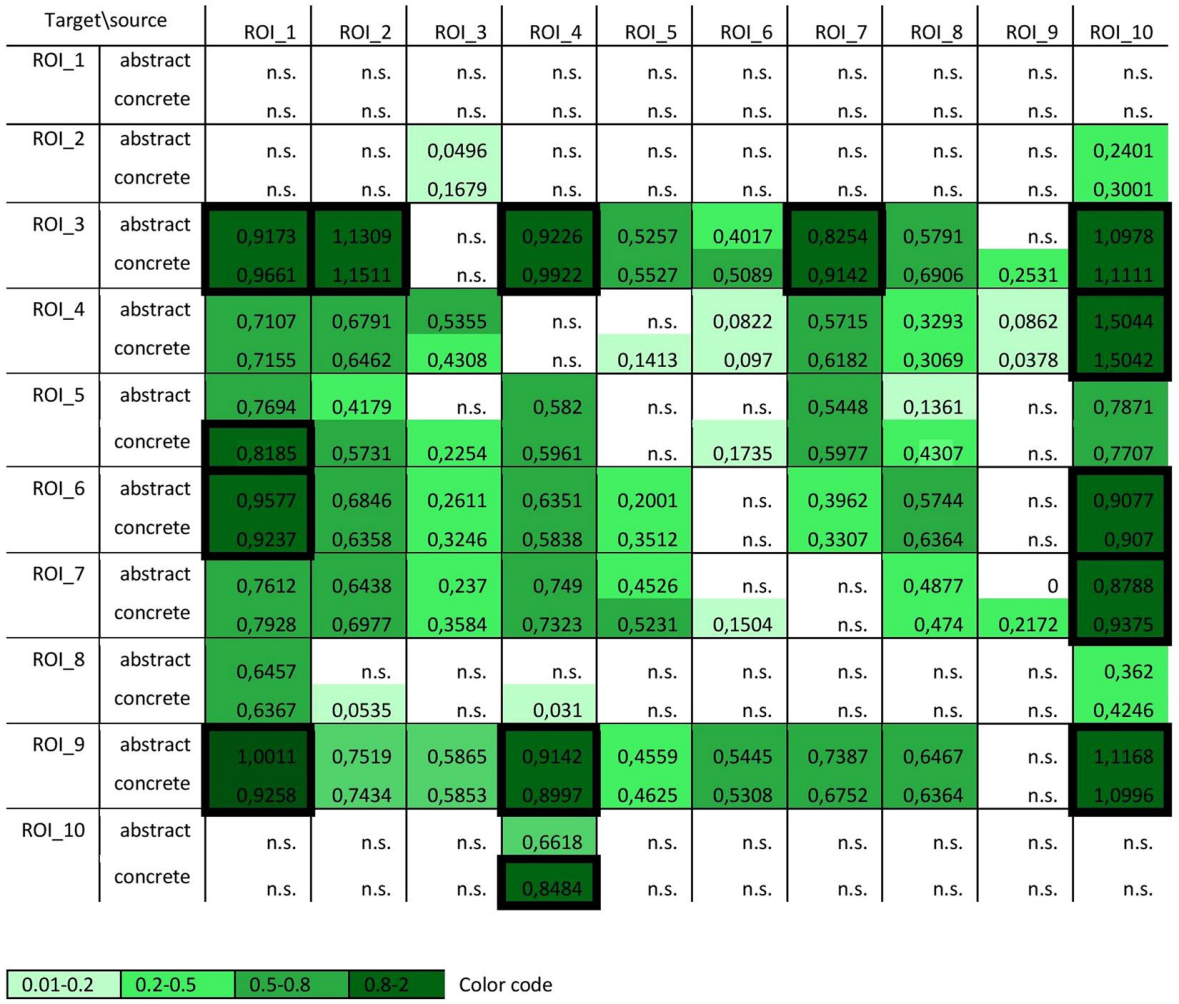
Effect sizes (Cohen’s d) for abstract and concrete trials for each ROI, given for significant connections (n.s. = not significant). Shades of green indicate magnitudes (0.01–0.2 small; 0.2–0.5 medium; 0.5–0.8 large; 0.8–2 very large or huge, colored from light to dark green, see color code below). All non-zero values are significant with p < 0.0001. The columns represent the senders of information, the rows the receivers.

We further estimated PDC values for the abstract and concrete trials of our 10 ROIs (see Materials and Methods) using the model estimation parameters described in supplementary material section B and the statistical analysis described in the section on Statistical analysis of Granger connectivity. Time-frequency plots of the PDC estimates can be found in supplementary material section C. For significant values of PDC, we estimated Cohen’s d effect sizes as shown in Fig. 4. Effect sizes are color-coded based on the rule of thumb^91^. We only discuss results with effect sizes larger than 0.8, as these are most likely to be replicated during the bootstrap resampling and Granger analyses with varying network sizes (shown in Fig. 4). Additionally, when testing for differences between our two conditions, we constrained our analysis only to the strongest connection (marked by accentuated connections with thick lines in Fig. 4) and corrected for multiple comparisons (number of connections) using Bonferroni’s correction. An exploratory analysis of these connections is reported in supplementary material section D.

**Figure 5:**
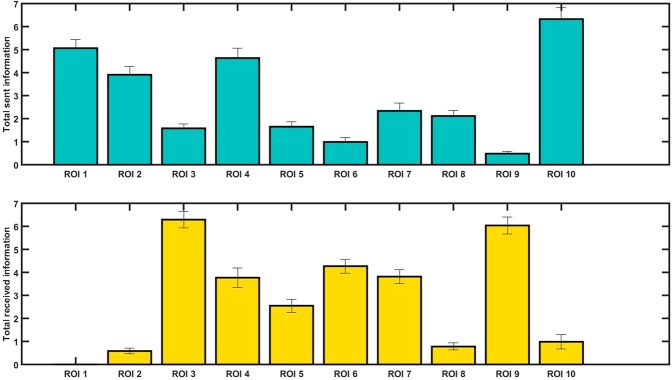
Total amount of outgoing (top, in blue) and incoming (bottom, in yellow) information flow for each region of interest. ROI 1 to 10 respectively represent the right superior occipital gyrus (1), the left orbitofrontal gyrus (2), the left anterior temporal lobe (3), the right anterior temporal lobe (4), the left posterior middle temporal gyrus (5), the right middle frontal gyrus (6), the left inferior temporal gyrus (7), the bihemispheric superior parietal lobule (8), the right middle and superior temporal gyri (9), and the right orbitofrontal gyrus (10).

For a concise overview of the connectivity pattern (for visualization purposes), we also plotted the results from Fig. 4 as a bar plot of averaged incoming and outgoing information flow. In Fig. 5, outgoing information flow for each region is represented as an average of all information coming *from* that region, which can also be seen in Fig. 4. Likewise, sent information is an average per region of information coming *into* a region, which can be gathered from the rows of Fig. 4. As Figs. 4 and 5 demonstrate, some regions play a bigger role in sending or receiving information compared to others. Results show that the right occipital lobe (ROI 1) is a main sender widely transferring information to the anterior parts of both hemispheres (Fig. 5) temporally and frontally, i.e. to left and right anterior temporal lobes (ROI 3 and 4), right middle frontal gyrus (ROI 6), left inferior temporal gyrus (ROI 7), bihemispheric superior parietal lobules (ROI 8) and right middle temporal gyrus (ROI 9). Similarly, the left and right orbitofrontal gyri (ROI 2 and 10) send information mostly to the same regions except for the superior parietal lobules (ROI 8). Furthermore, two regions largely receiving information are the left anterior temporal lobe (ROI 3, receiving from almost all areas except from the right middle and superior temporal gyri) and the right middle and superior temporal gyri (ROI 9, receiving from all areas). The remaining regions seem to be both senders and receivers of information.

##### Difference in information flow between abstract and concrete trials

Since our method of Granger analysis was based on time-varying autoregressive parameter estimation, we detected changes in connectivity strength over time and frequency. We investigated differences in information flow using the statistical analysis described in Materials and Methods (testing for significant difference in information flow between abstract and concrete trials).

##### Time window of significant difference in source activation (300–750 ms)

**Figure 6:**
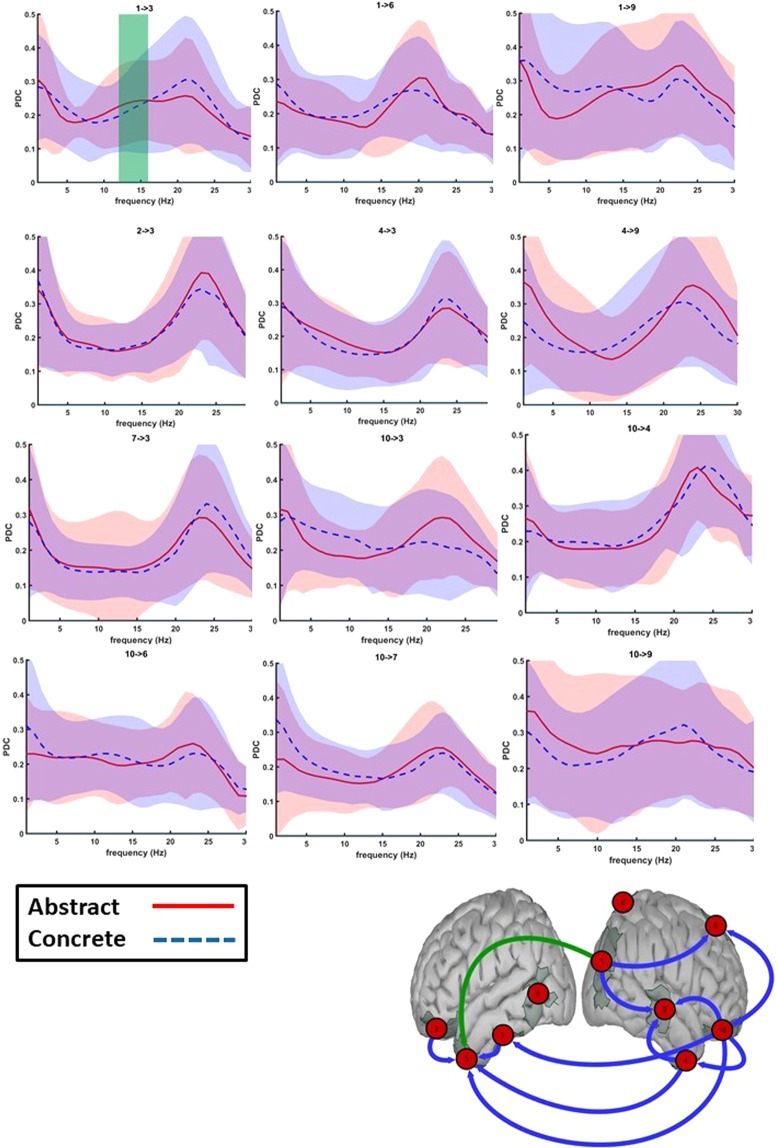
550–650 ms time window: Granger causality of strongest connections for both abstract and concrete trials shown in the frequency domain. Bottom figure: Connections in green display a significant difference between abstracts and concretes (for the frequency range highlighted in the green area in the top figure). Top figure: For the PDC values of abstracts and concretes, the 95% confidence interval is shown in shades of red and blue respectively.

**Figure 7:**
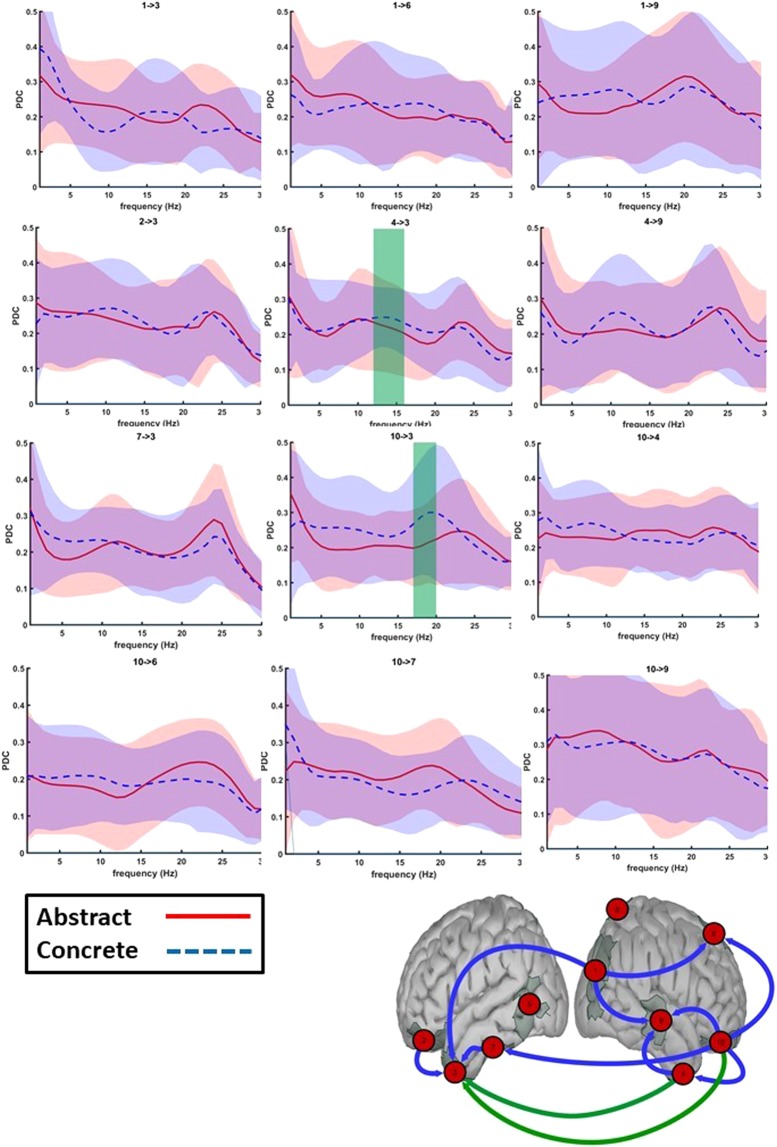
650–750 ms time window: Granger causality of strongest connections for both abstract and concrete trials shown in the frequency domain. Bottom figure: Connections in green display a significant difference between abstracts and concretes (for the frequency range highlighted in the green area in the top figure). Top figure: For the PDC values of abstracts and concretes, the 95% confidence interval is shown in shades of red and blue respectively.

Evaluating 100 ms time-bins in the given time-interval during which a difference between connectivity of abstract and concrete words was observed, we witnessed a statistically stronger information flow for abstract than concrete words from the right occipital lobe to the left anterior temporal lobe during the 550–650 ms time interval in the alpha band (p = 0.0008, effect size = 0.565 in the 8–13 Hz frequency range, see Fig. 6). Note that we consider the alpha band to range from 8–13 Hz and the beta band from 13–30 Hz. During a later time window (650–750 ms), several routes of information flow towards the left anterior temporal lobe exhibited stronger connections for concrete over abstract words (Fig. 7). These connections originated from the right anterior temporal lobe in the early beta band (p = 0.0092, effect size = 0.3609, frequency range = 12–16 Hz, 600–700 ms) and the right orbitofrontal gyrus in the beta band (p = 0.0046, effect size = 0.532, frequency range = 17–20 Hz, 650–750 ms). Interestingly, our exploratory analysis (for details, see supplementary material section D) additionally showed a stronger transfer of information in the beta band from the left posterior temporal and bihemispheric parietal lobules to the left anterior temporal lobe (p = 0.0036 and 0.0013 respectively, uncorrected). This demonstrates a general increase in beta band information flow towards the left anterior temporal lobe for concrete words during the 650–750 ms time window.

In addition to the above-mentioned analysis, we tested how the information flow varies in the time window which revealed a significant difference in activation between abstract and concrete words (the time span of 300–750 ms). Here, we found a general trend of increased alpha and early beta activity in the connections flowing towards the left anterior temporal lobe for concrete words (p < 0.0005 for all comparisons, Bonferroni corrected for the number of connection pairs).

## Discussion

The main goal of our study was to investigate and compare the network dynamics of abstract and concrete word processing. Our results on the scalp-level revealed a centro-frontal difference in EEG amplitudes between abstract and concrete words starting from around 300 ms after the words were presented^9,92^. Having moved on to investigate differences on the source level, we found that visual word processing does not entail a simple bottom-up process but includes both bottom-up and top-down connections. This was also suggested by Zhou *et al*.^93^, who investigated functional connectivity during text reading using fMRI and observed top-down regulation and prediction for the upcoming word. In our case, subjects were processing single words, which alleviates the amount of prediction. Furthermore, our results showed that reading activity mainly originates in the right extra-striate occipital cortex (visual cortex), ramifies to the right and left temporal lobes, medial superior parietal cortex and right middle frontal gyrus, and transfers itself further from the middle part of left inferior temporal gyrus and posterior temporal cortex to the left anterior temporal lobe. These connections encompass both ventral (occipito-temporal) and dorsal (occipito-parietal) streams of written-word processing. Observing information flow from the occipital cortex to both temporal and parietal cortices was not surprising, given that the extra-striate cortex is considered “a starting point” for both ventral and dorsal streams, respectively involved in semantic and phonological reading^94^. We also witnessed information flow between the visual cortex and the posterior temporal gyrus, the latter of which is known to be involved in lexical processing of reading^95^. Furthermore, we observed information transfer between the two anterior temporal lobes which, among others, are involved in semantic processing of word familiarity^96^.

Cognitive control during reading^97^ is exerted in areas of the ventral and dorsal streams. We observed an additional feedback system consisting of more anterior temporal areas (e.g. anterior temporal lobes), the left of which is believed to assume a semantic hub function^98^ sending information to posterior temporal regions assumedly regulating how the word form maps to its semantics. Overall, we can conclude that the right occipital lobe (bottom-up), and the bihemispheric orbitofrontal and right anterior temporal regions (top-down) are the strongest information senders, dispatching information to almost all other brain areas active during word processing. Areas mostly receiving information are the left anterior temporal and right middle temporal lobes, suggesting that the output of different processes converges in these areas (see Fig. 5). Several studies on general word and sentence reading uncovered similar characteristics of the network. For example, Schoffelen *et al*.^43^ used MEG data during sentence reading. Using Granger causality, they identified that the anterior temporal lobe on both hemispheres is a substantial receiver of information. While that was consistent with our results in the left anterior temporal lobe, in our study the right anterior temporal lobe seemed to be almost equally involved in sending and receiving information. Schoffelen *et al*. found that these areas receive information from the inferior frontal cortex, and the superior and middle temporal regions. Even though these results are very much in line with our findings, some differences can be observed given that their stimuli were sentences rather than single words. Other studies have shown an exceptional predominance of the occipito-temporal (OT) cortex in sending information^41^ and have, consistent with our findings, emphasized the importance of OT as the main entrance point from visual analysis to the language network. Furthermore, our study confirms that the medial, inferior and anterior temporal cortices are important for semantic processing, as previously suggested by Catani and Mesulam^99^.

### Comparison of connectivity patterns for abstract and concrete word comprehension

When comparing the connectivity structure of reading between abstract and concrete words, a higher connection strength during abstract word processing was only observed in the alpha band during the 550–650 ms time window. This can be explained by the presence of increased attention at this time for abstract words^100^ and the fact that abstract, compared to concrete, words have a more pronounced linguistic component^8^. Since abstract words are less imaginable they might additionally activate the anterior temporal lobe in the phase of early detection of the word category. It is important to note that the anterior temporal lobe is presumed to have a graded specialization where the superior part is predicted to be more active for abstract and the ventromedial part for concrete words. The spatial resolution of this study does, unfortunately, not allow for more fine-grained distinctions within the anterior temporal lobe^101^. This was the sole connection exhibiting a larger strength of connectivity for abstract word reading, as all remaining differences exerted a stronger connection during the reading of concrete words in the alpha and beta bands in a later time window. As the results showed, a stronger connection was observed during the time window of 650–750 ms from the right to left anterior temporal lobe in the alpha band and from the right orbitofrontal to the left anterior temporal lobe in the beta band. Additionally, our exploratory analysis for all time windows showed a consistently stronger network, predominantly in the beta band, during concrete word reading (see supplementary material section D). If the context availability theory would be extended in such a way as to account for connectivity, we hypothesize that abstract and concrete words would be processed in the same connectivity patterns but with differently weighted connections. As such, our findings would thus partly support the context availability theory. Abstract and concrete words are processed in bihemispheric, partially overlapping networks with the right hemisphere functioning as a sender and the left hemisphere as a receiver. The generally higher connectivity strength for concrete words can serve as a plausible explanation for the concreteness effect observed in behavioral studies (faster retrieval of concrete words).

A handful of studies have attempted to reveal differences in connectivity patterns of abstract and concrete words. In a series of studies, Weiss *et al*.^102–104^ measured the functional coupling using coherence analysis – a statistical measure for the correlation of signals within a certain frequency band. They found significantly higher coherence in the beta band (13–18 Hz) for concrete words, independent of presentation modality (visual or auditory), while the early alpha band (8–10 Hz) revealed identical coherence patterns. Despite both of them being spectral measures of connectivity, Granger causality based on PDC adopts, unlike coherence, a more stringent criterion for establishing information flow. Furthermore, coherence does not indicate the directionality of the flow. As such, there is not necessarily correspondence between coherence and causality (i.e. one does not imply the other)^105,106^. Similar to the studies conducted by Weiss, our exploratory analysis, despite the discrepancy in these methods, exhibited consistently higher beta band information flow that is stronger for concrete words (though unlike Weiss^102–104^, we also found differences in the alpha band).

Very recently, studies have specifically investigated connectivity differences between abstract and concrete single word reading^45,46^. Even though these studies relied on very different methods compared to ours, the results were more or less converging. In one study^45^, fMRI data was analyzed using group-ICA, uncovering an overall stronger connectivity for concrete words. In another study^47^, simultaneous MEG/EEG data was analyzed using dynamical causal modeling to reveal a modulation of the left anterior temporal lobe by word concreteness starting as early as 150 ms (but also during later stages). Moreover, they found a stronger connection between the left anterior temporal lobe and the right orbitofrontal cortex for abstract words, contemplating that this might be a result of abstract words being rated as more emotional (higher valence) than concrete words. Since we controlled for the affective dimensions of valence, activity and potency, we, unsurprisingly, did not make the same observations. Importantly, the above-mentioned studies employed an explicit concreteness task, i.e. participants were aware of the purpose of the study, which was shown to increase the evaluated concreteness effect^9^. Our participants, however, were instructed silently to read the words for comprehension and press a button upon seeing the arbitrary category “color” (implicit categorization task) after which the corresponding data was removed. Therefore, we believe that the EEG patterns evoked by our paradigm reflect the processing of words in more natural conditions.

### Suggestions for future research and limitations of the study

In the current study, we used a multivariate, time-varying adaptation of Granger causality on source localized EEG data in order to investigate the spatial, spectral and temporal dynamics of the information flow during single word reading. Such a model is computationally complex as it requires many data points to be trained. The complexity of a multivariate model is known to be relative to the number of variables to the power of two multiplied by the order of the model (*O*(*m*^2^*p*)). Therefore, even a small number of variables can dramatically increase the need for more trials to ensure a good fit. We did not attempt to create a large network with many regions of interest even though other regions reported in the literature could have potentially been of interest to our study. We also do not recommend analyzing GC with only a portion of all ROIs to decrease computational complexity. As all causal factors need to be incorporated in the model, Granger Causality may produce misleading results when the true relationship involves more variables than those that have been selected^107^. In our case, the network constituted all regions with either common or differential neural activation between our two paradigms. We can, however, not preclude that not all ROIs playing a causal role in neural dynamics have been successfully identified, as some may have been downregulated during task performance^37^.

Furthermore, along with high model complexity, estimating a large network can also be problematic in terms of the multiple comparisons problem. Therefore, we limited our analysis to the alpha and beta frequency bands during the time windows for which significant differences in activation were observed. In our exploratory analysis, reported in supplementary material section D, we analyzed all consecutive time windows. It is important to note, though, that we do not suggest that there be a simple relationship between difference in activation and difference in connectivity. In fact, an exploratory analysis has demonstrated connectivity differences during earlier time windows. Even during the selected time windows, areas showing a difference in activity were not necessarily those involved in connectivity differences between conditions. An interesting future study would be to investigate the interaction between local measures of activation and connectivity. Another would be to explore an adaptive model order. In our current parameter estimation, the model order was kept constant. However, it is very well possible that some connections have faster information flow than others, therefore requiring a smaller time lag when assessing their connectivity. Knowing the optimal model order for each connection could indicate a difference in the speed of information transfer for particular routes in the network and might be able to explain the faster reaction time and retrieval of concrete words.

Finally, in this study we have limited ourselves to sources localized on the cortical surface even though many subcortical structures such as the thalamus and some parts of the basal ganglia are suggested to contribute to language processing^108,109^. Despite the fact that it is still unclear how activity from deeper structures can be detected by means of EEG source reconstruction, more studies are now claiming that activity from subcortical structures can reliably be estimated using high-density EEG^110,111^. How subcortical structures are posited in the Granger network of information flow during word processing is another question for future research.

## Conclusion

We have provided evidence for directed interactions between cortical regions in the language network of the human brain during single word processing and have investigated differences in activation and interactions of information flow when reading abstract versus concrete words. Our state-of-the art approach allowed us to investigate patterns of information flow in the spatial, temporal as well as spectral domains. Our results showed a network of interactions involving regions located along both ventral and dorsal pathways where areas at both ends of the stream were mainly involved in sending information (right superior occipital lobe and orbitofrontal gyrus on both hemispheres). By contrast, areas which were largely recipients of information were the left anterior temporal lobe and right middle temporal gyrus. Further comparison between network interactions during abstract and concrete single word reading revealed increased information flow (stronger network) for both beta and alpha bands during processing of concrete words around 550–750 ms and an increase in alpha interactions for abstract words in the 550–650 ms time window. These differences all concerned information transfer routes flowing to the left anterior temporal lobe (ATL), suggesting that this area is largely involved in the processing of abstract words. Given the previous suggestions that the left ATL is considered a semantic hub^98^ and our observations of its stronger connections with other brain regions for concrete compared to abstract words, we propose that this information flow reflects the matching of the lexical representation of the concrete words with its semantic knowledge. Further exploratory analysis revealed generally higher connectivity during concrete word processing. These findings suggest that abstract and concrete words are processed in partially overlapping networks even though the strength of their connectivity exhibits different spectral and temporal properties.

## Supplementary information

Supplementary Material.

## Data Availability

The datasets generated during and/or analyzed during the current study are available from the corresponding author on reasonable request.
