# Supplementary material for: Overlapping connectivity patterns during semantic processing of abstract and concrete words revealed with multivariate Granger Causality analysis
Source: Sci Rep. 2020 Feb 18;10:2803. doi: 10.1038/s41598-020-59473-7 (PMC7028761; doi:10.1038/s41598-020-59473-7)
Supplement: Supplementary file 1 — Supplementary Material. [file 41598_2020_59473_MOESM1_ESM.pdf]

# Supplementary Material

**Overlapping connectivity patterns during semantic processing of abstract and concrete words revealed with multivariate Granger Causality analysis**

Mansoureh Fahimi Hnazaee*[1], Elvira Khachatryan,[1] Sahar Chehrazad[2], Ana Kotarcic[3], Miet De Letter[4], Marc M. Van Hulle[1]

[1] Laboratory for Neuro- and Psychophysiology, Department of Neurosciences, KU Leuven, Leuven, Belgium

[2]Numerical Analysis and Applied Mathematics Section, Department of Computer Science, KU Leuven, Leuven, Belgium

[3]Center for the Historiography of Linguistics, Department of Comparative, Historical and Applied Linguistics, KU Leuven, Leuven, Belgium

[4]Medicine and Health Sciences, Department of Rehabilitation Sciences, UGent, Gent, Belgium

*Corresponding author

**Appendix A.**

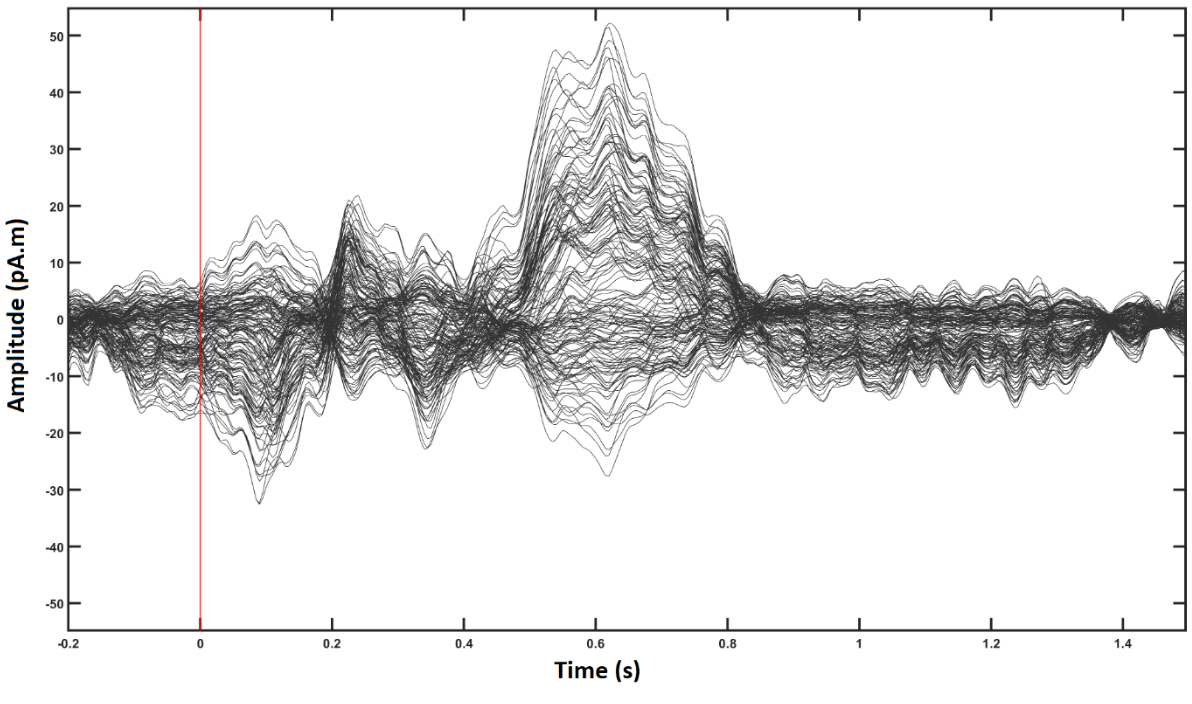


*Figure S1 Time series within ROI 3 (left anterior temporal lobe). Time series are qualitatively similar; depending on their location on the sulci/gyri dipole orientation is opposite for some time series. Orientation does not affect the outcome of Granger Causality.*

**Appendix B.** Autoregressive model parameter estimation

To tune the parameters and validate an autoregressive model, several criteria have been proposed:

a) The Bayesian Information Criterion (BIC) [1],
$\Phi_{M,N}(p) = \ln[\det(\sum_N(p))] + \frac{\ln(N)}{N} M^2 p$
where $M$ denotes the number of variables, $N$ the number of time samples per trial and $\sum_N(p)$ the variance of the prediction error for model order $p$.

b) The whiteness test which necessitates that the residuals be uncorrelated white noise. For our study, we used the Li-McLeod Portmanteau test modified for multi-variate models [2].
c) The stability test which requires the eigenvalues of the autoregressive parameters to be smaller than 1.
d) The percent consistency which is a comparison of the correlation vector of the real and reconstructed data using the autoregressive parameters [3].

After performing a parameter exploration, we settled for a model order of 14 (corresponding to a time lag of 70ms) and an adaptation coefficient of 0.01 which passed the whiteness test and is also in line with simulation studies on the multi-trial General Linear Kalman filter [4,5].

Some studies further validate the model order selection by comparing the power spectrum of the signal using a non-parametric Welch and parametric Burg method [6]. In our parameter exploration, we realized that the criteria described above do not converge to the same optimal parameters. For example, it has been previously mentioned that increasing the adaptation coefficient increases the speed of adaptation at the cost of losing smoothness of the estimates [4]. We also observed that a high adaptation coefficient increases the correlation of the residuals and decreases stability. However, a lower adaptation coefficient decreased the percent consistency. The parameter exploration of a sample subject is shown in figure S2.

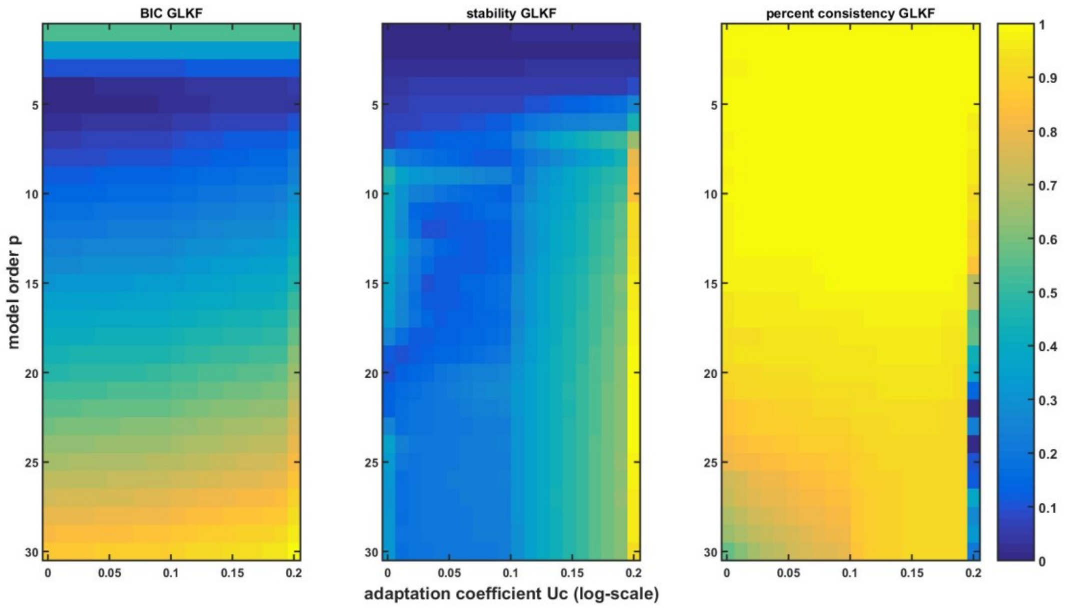


*Figure S2 Parameter exploration results of three different criteria (BIC, stability, percent consistency). Results are normalized between 0 and 1. Note that for parameter estimation, BIC and stability need to be minimized for model validation and maximized for percent consistency.*

As can be seen in figure S2, there is always a trade-off between the different criteria defined in the literature. However, it should also be noted that these criteria were designed for purposes other than Granger connectivity. In our case, the most important criterion for Granger connectivity is consistency. Therefore, we performed a bootstrapping analysis of the Granger causality (described in section Statistical analysis of Granger connectivity).

**Appendix C.** Partial Directed Coherence results

The results for abstract trials are shown below.

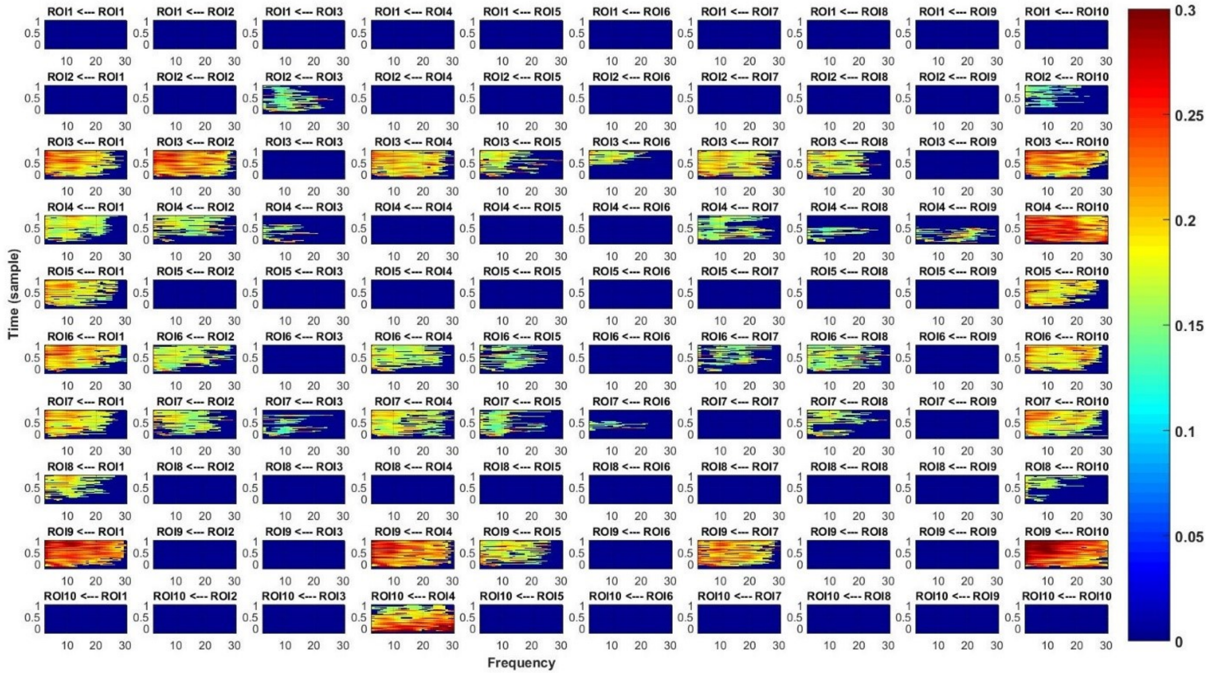


*Figure S3 Within-subject cluster-based non-parametric permutation test with 10,000 iterations for abstract trials. All shown results are significant for p<0.0001.*

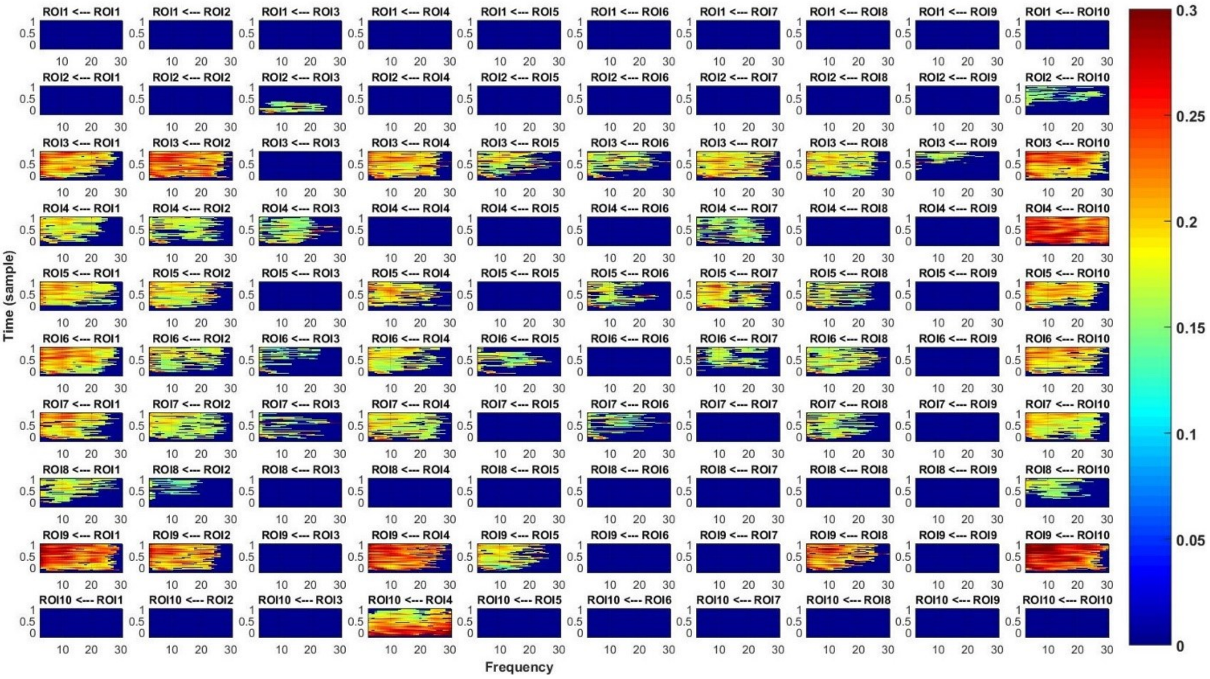


*Figure S4 Within-subject cluster-based non-parametric permutation test 10,000 iterations for concrete trials. All shown results are significant for p<0.0001.*

**Appendix D.** Exploratory analysis of statistically significant connections

An exploratory analysis of abstract versus concrete words was performed for every 100ms and for all connections with an effect size above 0.8. The p value was set to 0.01 (uncorrected). We did not correct for multiple comparisons over time and connection pairs. However, similar to the main results we only considered results which were significant for a frequency band of minimum 3Hz. Additionally, results were only considered between 5 and 25 Hz, as we were apprehensive of edge artifacts outside this frequency range.

*Table S1 p represents p value (uncorrected), d Cohen's d effect size and f frequency range of significance*

| | *Abstract>Concrete* | | *Concrete>Abstract* | |
|---|---|---|---|---|
| Time \Frequency | *Alpha* | *Beta* | *Alpha* | *Beta* |
| *100-200ms* | n.s | n.s | n.s | Left anterior temporal to right anterior temporal lobe ROI 3->ROI 4 (p=0.0079, d=0.432 f=12-15Hz)<br><br>Right anterior temporal lobe to right middle temporal lobe ROI 4-> ROI 9 (p=0.0035, d=0.593, f=20-23Hz) |
| *200-300ms* | n.s | n.s | Bihemispheric superior parietal to right middle frontal gyrus ROI 8-> ROI 6 (p=0.0079 d=0.473, f=11-13Hz) | n.s |
| *300-400ms* | n.s | n.s | | Left posterior middle temporal to left orbitofrontal gyrus ROI 5 -> ROI 2 (p=0.0035, d=0.629, f=21-24Hz) |
| *400-500ms* | n.s | n.s | Left inferior temporal to right middle temporal gyrus ROI 7 -> ROI 9 (p=0.0025 d=0.2889 f=10-12Hz) | Right superior occipital to bihemispheric superior parietal ROI 1 -> ROI 8 (p=0.0075, d=0.640 f=16-18Hz) |
| *500-600ms* | Right superior occipital lobe to left anterior temporal lobe ROI 1 -> ROI 3 (p=0.0090, d=0.482, f=11-13Hz) (for 550-650ms: p=0.0008, d=0.565, f=8-13Hz) | | n.s | n.s |
| *600-700ms* | n.s | n.s | n.s | Left posterior temporal to left anterior temporal lobe ROI 5 -> ROI 3 (p=0.0036, d=0.371, f=19-21Hz) |

| | | | | |
|---|---|---|---|---|
| | | | | Right orbitofrontal gyrus to left anterior temporal lobe ROI 10 -> ROI 3 (p=0.0046, d=0.556, f=18-20Hz) |
| *700-800ms* | n.s | n.s | n.s | Bihemispheric superior parietal to left anterior temporal lobe ROI 8 -> ROI 3 (p=0.0013, d=0.454, f=23-25Hz) |

**Appendix E.** Inter-subject variability of connection pairs

In order to gauge the effect of inter-subject variability, we separated all time-frequency data points per subject into significant and non-significant connection pairs. The histogram of the standard deviation and the mean over subjects are shown in figure S5. As can be seen, the standard deviation of non-significant connections was much smaller than that of the significant connections. This suggests that when a connection pair is not significant, it is fairly consistent between subjects. However, the significant connections vary across subjects.

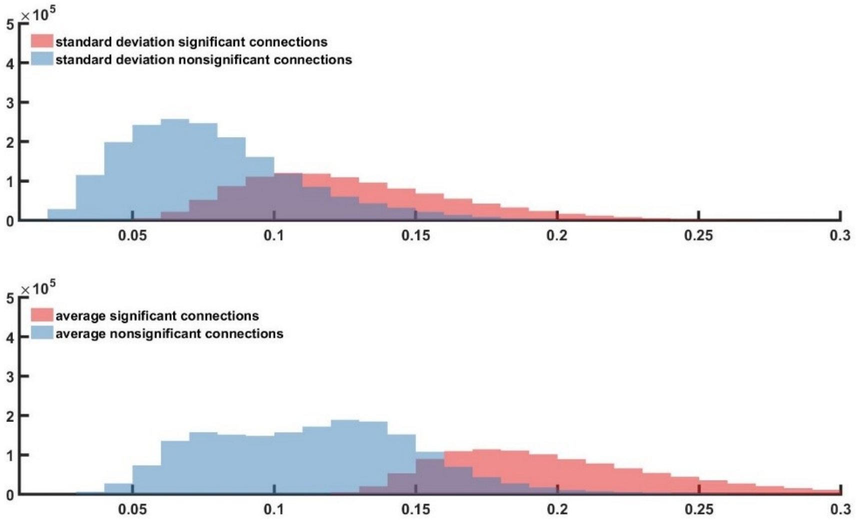


*Figure S5 Standard deviation (top) and mean (bottom) of significant and nonsignificant connections*

**Appendix F.** list of target stimuli presented during the task

Abstract concepts

| Active | Passive | Positive | Negative | Strong | Weak |
|---|---|---|---|---|---|
| lastig | doodmoe | ijver | ergernis | woest | armoede |
| assertief | rustig | artistiek | furieus | genie | preuts |
| opluchting | passief | psyche | dwingend | probleem | toegeeflijk |
| sadist | pensioen | verheugd | lafaard | triomfantelijk | bangerik |
| fascinatie | doofpot | genadig | lafhartig | charmant | passief |
| schrik | sleur | ondernemend | verward | wellevend | hopeloosheid |
| verdacht | machteloos | oprecht | schuld | durf | gedwee |
| ambitie | pauze | jong | wanhopig | grof | lamlendig |
| ijzersterk | moedeloos | machtig | passief | rechtschapen | overgave |
| bemind | werkloosheid | vastbesloten | belabberd | positief | schriel |
| opgelucht | ingehouden | voordeel | verlies | overheersend | onvolwassen |
| potentie | lusteloos | plechtig | onaangenaam | dierbaar | zwakkeling |
| vijandig | uitgedoofd | opmerkelijk | sleur | aanzienlijk | treurigheid |
| wandaad | depressief | beroemd | nervositeit | doorbraak | onmacht |
| progressief | volgzaam | verdraagzaam | sukkelig | magisch | timide |
| irrationeel | ontmoedigd | genie | misbruik | zegen | nietig |
| vermakelijk | teruggetrokken | fraai | bedreiging | god | hulpeloos |
| manipulatief | apathisch | ingetogen | onpraktisch | meedogenloos | onbeholpen |
| knap | eenvoudig | toekomst | ziekelijk | overtuigd | onderdanig |
| verkwister | eenzaamheid | welvaart | zondig | zuiver | volgzaam |
| ongerust | gedwee | zachtmoedig | ondankbaar | limiet | neerslachtig |
| beledigd | hopeloosheid | vriend | dictator | optimistisch | gebrekkig |
| schoonheid | werkloos | voorzichtig | incompetent | nieuwsgierig | gedienstig |
| dwangsom | kalmte | realistisch | roddel | zelfgenoegzaam | mislukking |
| ervaren | neerslachtig | nadenkend | heibel | verwonderd | onbekwaam |
| irriterend | apathie | schoonheid | machteloos | bedreven | verslagen |
| bedreven | depressie | superieur | besluiteloos | uitdagend | wanhopig |
| kans | zwakkeling | bemind | verdriet | verantwoordelijk | kwetsbaar |
| haat | zorgeloos | hartstochtelijk | verkeerd | stabiel | weerloos |
| broederschap | berusting | enthousiast | vrees | humeur | ondeskundig |
| potent | schuchter | degelijk | vloek | spontaan | nederig |
| bemoeiziek | bedaard | geanimeerd | ongelukkig | drang | lafaard |
| gefrustreerd | doelloos | mogelijkheid | schraal | cynisch | schamel |
| gloednieuw | afwachtend | stoer | boosaardig | verraad | teruggetrokken |
| ondernemend | energieloos | lyrisch | onrustig | eigenzinnig | verloren |
| vooruitstrevend | berustend | intellectueel | riskant | zelfbewust | onzekerheid |
| panisch | onverschillig | weldaad | mislukking | standvastig | sukkelig |
| jong | somber | getalenteerd | rotzak | competitief | verlegenheid |
| vrijgevig | vredig | vermakelijk | pover | opvallend | armzalig |
| contact | incompetent | ijverig | ontmoedigd | crisis | onderworpen |
| zelfvoldaan | comfort | tevredenheid | wanhoop | bruto | meegaand |
| opgewektheid | introvert | tactvol | illegaal | robuust | verlegen |
| illegaal | onderworpen | fascinatie | incest | bazig | werkloos |
| aandachtig | hulpeloos | wijs | somber | ongehoorzaam | bedeesd |
| trots | verveling | invloed | krachteloos | perversie | nalatig |
| avontuurlijk | liefdeloos | legaal | droevig | gunst | overgevoelig |
| rebels | onemotioneel | winst | ontevreden | bruusk | reddeloos |
| afschuw | bescheiden | korting | hebzuchtig | invloedrijk | depressie |
| extreem | zachtaardig | frivool | bemoeiziek | extravert | moedeloos |
| welvaart | bedeesd | galant | bedrieglijk | belasting | twijfelachtig |

Concrete concepts

| Active | Passive | Positive | Negative | Strong | Weak |
|---|---|---|---|---|---|
| sheriff | strand | uurwerk | rugpijn | standbeeld | vlucht |
| station | gordijn | aquarium | gevecht | hersens | rolstoel |
| bruiloft | sluier | basketbal | karkas | vulkaan | pleister |
| trompet | kapel | medaille | luizen | scepter | gebroken |
| dolfijn | tombe | fornuis | termiet | ontploffing | vuilnisbak |
| buskruit | bijbel | garnaal | koorts | spinazie | lippenstift |
| bazooka | slaapzaal | palmboom | drijfzand | schavot | gehandicapt |
| gangster | fluweel | schaats | knobbel | dwangbuis | vlinder |
| bordeel | kerkhof | speelgoed | maagzweer | patroon | hoofddoek |
| carnaval | fauteuil | station | geraamte | leerkracht | konijn |
| infectie | hoofddoek | hamster | aardbeving | drijfzand | kalkoen |
| sprinkhaan | behangpapier | gletsjer | ongeval | vliegtuig | tomaat |
| kapitein | zitplaats | cocktail | dronkaard | gletsjer | perzik |
| maagzweer | hoofdsteun | medicijn | granaat | gasmasker | schaduw |
| scrotum | schaduw | sleutel | cycloon | verkrachting | butler |
| voertuig | begrafenis | scharnier | stront | doelpunt | yoghurt |
| fanfare | plafond | kleding | stinkdier | aardbeving | kreupel |
| frisbee | greppel | regenboog | kreupel | cycloon | linnen |
| gijzelaar | klooster | framboos | ravijn | ongeval | kelner |
| stinkdier | standbeeld | gespierd | kleurloos | basketbal | kikker |
| vrachtwagen | pastoor | kalkoen | scheet | maagzweer | vlieg |
| bliksem | doodskist | kapitein | ontploffing | spijker | dronkaard |
| leerkracht | tapijt | yoghurt | skelet | machine | zacht |
| vuurwerk | vloer | olifant | ziekenhuis | knuppel | karton |
| ontploffing | steen | hazelnoot | bivakmuts | glimlach | dwerg |
| vulkaan | | meester | rolstoel | voetbal | |
| schreeuw | | kostuum | beerput | scooter | |
| koekoek | | trapezium | oorworm | ziekenhuis | |
| schavot | | smaragd | doodskist | gespierd | |
| streling | | beschuit | duister | carnaval | |
| kernbom | | vlinder | keelpijn | schaats | |
| naaimachine | | tentoonstelling | buikpijn | dansvloer | |
| hagedis | | zonneschijn | knuppel | gezicht | |
| gasmasker | | planeet | bordeel | kostuum | |
| kakkerlak | | koekoek | infectie | revolver | |
| basketbal | | laurier | stortbui | stortbui | |
| knipoog | | lippenstift | tandpijn | politie | |
| olifant | | spreeuw | orkaan | knokkel | |
| revolver | | spieren | lawaaierig | fabriek | |
| snotneus | | luipaard | verkrachting | gangster | |
| kleuter | | fanfare | gebroken | schipbreuk | |
| adelaar | | fluweel | lawaai | pistool | |
| verkrachting | | vaandel | vergif | doelwit | |
| confetti | | steiger | lawine | buskruit | |
| luipaard | | leerkracht | gijzelaar | tandpijn | |
| roofmoord | | prairie | kloten | bazooka | |
| vloedgolf | | applaus | vloedgolf | termiet | |
| roofdier | | orgasme | pooier | tequila | |
| hamster | | feestmaal | pissebed | steiger | |
| knuppel | | makreel | dwangbuis | begrafenis | |

**References**


1. Schwarz, G. Estimating the dimension of a model. *Ann. Stat.* **6**, 461–464 (1978).
2. Li, W. K. & McLeod, A. I. Distribution of the Residual Autocorrelations in Multivariate ARMA Time Series Models. *J. R. Stat. Soc.* **43**, 231–239 (1981).
3. Ding, M., Bressler, S. L., Yang, W. & Liang, H. Short-window spectral analysis of cortical event-related potentials by adaptive multivariate autoregressive modeling: data preprocessing, model validation, and variability assessment. *Biol. Cybern.* **83**, 35–45 (2000).
4. Pagnotta, M. F. & Plomp, G. Time-varying MVAR algorithms for directed connectivity analysis: Critical comparison in simulations and benchmark EEG data. *PLoS One* **13**, e0198846 (2018).
5. Ghumare, E. G., Schrooten, M., Vandenberghe, R. & Dupont, P. A Time-Varying Connectivity Analysis from Distributed EEG Sources: A Simulation Study. *Brain Topogr.* **31**, 721–737 (2018).
6. Mierlo, P. Van *et al.* Functional brain connectivity from EEG in epilepsy: Seizure prediction and epileptogenic focus localization. *Prog. Neurobiol.* **121**, 19–35 (2014).
